# Biomedical Applications of an Ultra-Sensitive Surface Plasmon Resonance Biosensor Based on Smart MXene Quantum Dots (SMQDs)

**DOI:** 10.3390/bios12090743

**Published:** 2022-09-09

**Authors:** Seyyed Mojtaba Mousavi, Seyyed Alireza Hashemi, Masoomeh Yari Kalashgrani, Vahid Rahmanian, Ahmad Gholami, Wei-Hung Chiang, Chin Wei Lai

**Affiliations:** 1 Chemical Engineering Department, National Taiwan University of Science and Technology, Taipei City 106335, Taiwan; 2 Nano-Materials and Polymer Nano-Composites Laboratory, School of Engineering, University of British Columbia, Kelowna, BC V1V 1V7, Canada; 3 The Center of Biotechnology Research, Shiraz University of Medical Science, Shiraz 71468-64685, Iran; 4 The Centre of Molecular and Macromolecular Studies, Polish Academy of Sciences, Sienkiewicza 112, 90-363 Lodz, Poland; 5 Nanotechnology & Catalysis Research Centre (NANOCAT), Level 3, Block A, Institute for Advanced Studies (IAS), Universiti Malaya (MU), Kuala Lumpur 50603, Malaysia

**Keywords:** surface plasmon resonance, biosensor, smart MXene quantum dots, biomedical

## Abstract

In today’s world, the use of biosensors occupies a special place in a variety of fields such as agriculture and industry. New biosensor technologies can identify biological compounds accurately and quickly. One of these technologies is the phenomenon of surface plasmon resonance (SPR) in the development of biosensors based on their optical properties, which allow for very sensitive and specific measurements of biomolecules without time delay. Therefore, various nanomaterials have been introduced for the development of SPR biosensors to achieve a high degree of selectivity and sensitivity. The diagnosis of deadly diseases such as cancer depends on the use of nanotechnology. Smart MXene quantum dots (SMQDs), a new class of nanomaterials that are developing at a rapid pace, are perfect for the development of SPR biosensors due to their many advantageous properties. Moreover, SMQDs are two-dimensional (2D) inorganic segments with a limited number of atomic layers that exhibit excellent properties such as high conductivity, plasmonic, and optical properties. Therefore, SMQDs, with their unique properties, are promising contenders for biomedicine, including cancer diagnosis/treatment, biological sensing/imaging, antigen detection, etc. In this review, SPR biosensors based on SMQDs applied in biomedical applications are discussed. To achieve this goal, an introduction to SPR, SPR biosensors, and SMQDs (including their structure, surface functional groups, synthesis, and properties) is given first; then, the fabrication of hybrid nanoparticles (NPs) based on SMQDs and the biomedical applications of SMQDs are discussed. In the next step, SPR biosensors based on SMQDs and advanced 2D SMQDs-based nanobiosensors as ultrasensitive detection tools are presented. This review proposes the use of SMQDs for the improvement of SPR biosensors with high selectivity and sensitivity for biomedical applications.

## 1. Introduction

The rapid improvement of technology, in line with the growing needs of modern societies in today’s world, has paved the way for the development of the factors involved and lifestyles. Sensors are devices that help researchers in various fields such as industry, agriculture, etc. to achieve certain goals by measuring predetermined parameters. Biosensors are a special category of sensors used to study and detect chemical and biological parameters. The diagnosis of diseases, the discovery of new drugs, and the identification of contamination by biological factors such as DNA, proteins, antibodies, enzymes, and viruses are performed by systems called biosensors. Based on their operation, these sensors are classified into mechanical, chemical, electrical, and optical groups. Apart from these, optical biosensors are divided into labeled and label-free sensors [1,2,3]. Optical biosensors have several advantages, including high sensitivity and insensitivity to electromagnetic interference. Optical biosensors have also covered a wide range of substrates, including SPR, localized surface plasmon resonance (LSPR), interferometers, ring amplifiers, etc. [4]. There are certain types of biosensors that use light sources and light guidance based on different methods to achieve detection and detection objectives. One of the most common types of these sensors are biosensors that use plasmonic sensing in the design of their structure and are referred to as surface plasmon resonance biosensors. SPR biosensors stimulate the phenomenon of the oscillation of electrons in the metal-dielectric junction when their rate of motion matches the rate of motion of the incident light. This category of sensors is of interest to many researchers and scientists in this field due to their small size and optimal sensitivity [5,6]. Therefore, the development of SPR biosensors can be an important area of research to find chemical and biological substances that cause diseases or have negative consequences [7,8,9]. One of the most important aspects in the development of SPR biosensors may be the accurate detection of ‘target molecules’ to prevent the occurrence of disease and facilitate early medical therapy. Ultimately, this will accelerate therapeutic efficacy [9,10,11]. To this end, high selectivity and sensitivity are the most important properties to be considered in the development of SPR biosensors. Numerous nanomaterials, including metal NP and transition metal dichalcogenide (TMD) NP, are being investigated for the development of SPR biosensors [12,13,14,15]. Although metal NPs have been more commonly used in the past [16,17,18,19], after the discovery of carbon nanomaterials, such as graphene, these nanomaterials showed a more efficient performance than current metal NPs [20,21,22,23], and their biocompatibility can render them suitable for monitoring cell-size conditions [24,25,26]. However, the development of new nanomaterials for SPR biosensors is possible because the demand for nanomaterials with exceptional properties and efficient performance is constantly increasing. 2D nanomaterials, such as smart MXene, are becoming increasingly popular due to their special properties, such as physical, electrical, and chemical properties [27,28]. The term ‘smart MXene’ has been used by a number of researchers for MXene-based hybrid materials, indicating their unique application-related properties. Such unique compounds make MXene a potential candidate for the fabrication of transparent conductors. MXenes offer transmittance up to 95% in the visible and UV regions with very low sheet resistance (up to 0.01 kO per square). Due to their excellent mechanical properties and tunable optical properties, MXenes can be used as transparent conductive electrodes for touchscreen applications, various sensors, light-emitting diodes, and flexible displays [29,30,31]. Among the nanomaterials for SPR biosensors, SMQDs attract much attention due to their great potential and exclusive properties in developing SPR biosensors [32,33,34]. SMQDs are 2D inorganic compounds composed of transition metal carbides and possess a significant atomic layer thickness. Their exceptional properties include high conductivity and plasmonic and optical qualities [35,36,37,38]. SMQDs can be used in biomedical applications due to their biocompatibility. [39,40,41,42]. This new nanomaterial is now the best option for the development of SPR biosensors in biomedical applications, based on the present research to improve SPR biosensors. The main objective of this review is an ultra-sensitive plasmon resonance nano biosensor on a surface based on SMQDs for biomedical applications. This article is divided into three topics: an introduction to SPR and SPR biosensors; explanation and characteristics of SMQDs; and SPR biosensors based on SMQDs. Accordingly, this review clearly presents the characteristics of SMQDs for the development of SPR biosensors and their biomedical applications. In summary, the team believes that this article can highlight current research directions as well as ways to utilize SMQDs for the efficient improvement of SPR biosensors with a high selectivity and sensitivity in biomedical applications.

## 2. SPR

**Figure 1:**
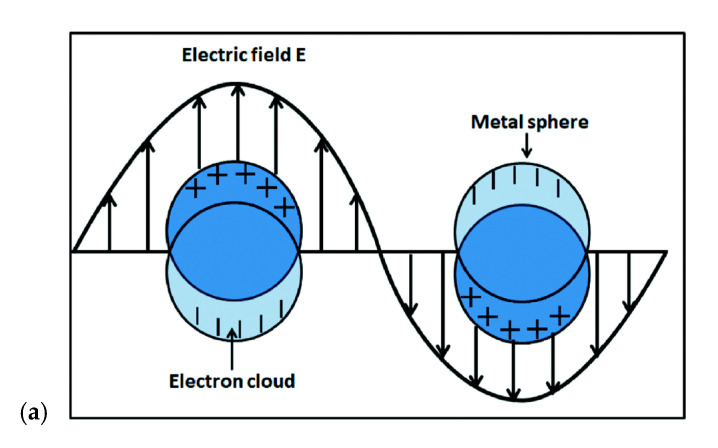

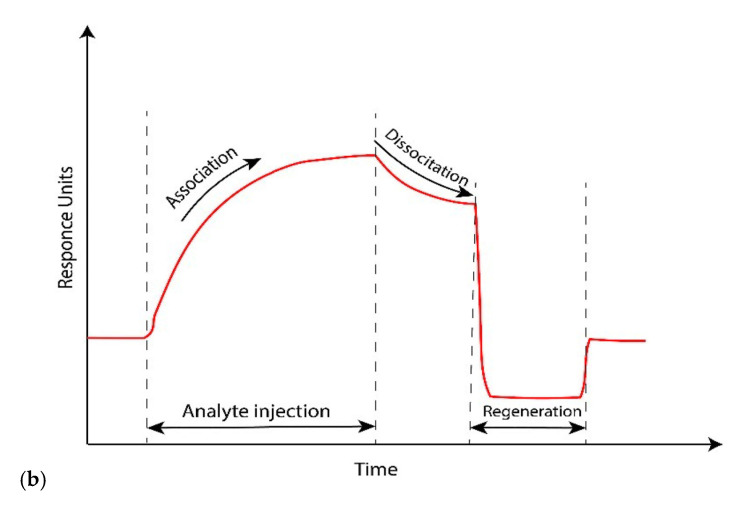
(**a**) Schematic of the surface plasmon (electronic cloud) resonated due to the electric field. (Reprinted with permission [51]. Copyright © 2016, The Author(s). Licensee: IntechOpen.) (**b**) Steps in a typical SPR method. (Reprinted with permission [49] Copyright © 2020 by the authors. Licensee MDPI, Basel, Switzerland).

SPR refers to the collective oscillations of electrons on the surface of metal nanostructures that occur in response to an external stimulus such as light or a charge. When the particle size reaches the order of nanometers, the electron can spontaneously accelerate on the surface of the particle and absorb electromagnetic waves of a certain wavelength; a schematic of this phenomenon is shown in Figure 1a [43,44,45]. The solutions of nanoparticles of rare metals such as silver and gold (which have a high conductivity) often show a strong absorption band in the visible spectrum. When the solution of these nanoparticles with the same size range is exposed to electromagnetic radiation, part of the radiation is scattered, and part of it is accelerated by the free electrons of the nanoparticles; therefore, in this phenomenon, certain frequencies are absorbed, resulting in an enhancement of the electron resonance and appearing as a strong peak in the visible region. The shape and frequency of the resonance spectrum depend on the size, shape, and distance between the nanoparticles and their dielectric properties but, most importantly, on the dielectric properties of the environment in which the nanoparticles are located [46,47,48]. Figure 1b shows the steps of a typical SPR method. Each sensor-level measurement begins with the selection of a suitable buffer solution, which is the most basic task before starting the association process. At this stage, the sensor surface contains active ligands ready to accept the target analytes. By injecting the solution containing the analytes, the association cycle begins. If the correct ligands are not selected, special bonds can form between the ligands and the analytes after the solution has passed, leading to the instability and detachment of the ligands from the surface. In this step, the kinetic energy resulting from the interaction of analytes and ligands is measured in real time. In the next step, a solution is brought into contact with the sensor surface to regenerate the initial state. As shown in Figure 1b, this step destroys the non-specific binding elements so that the mass accumulated on the surface can be recovered from the sensor reaction. At this stage, analyte dissociation also begins, and the kinetic energy of the dissociation process can be studied. Finally, the regeneration solution is injected, breaking the link between ligands and analytes. If the ligands are properly placed on the surface, they will remain on the surface after passing the regeneration solution as the analytes are gradually removed [49,50].

### Biosensor Using SPR

**Figure 2:**
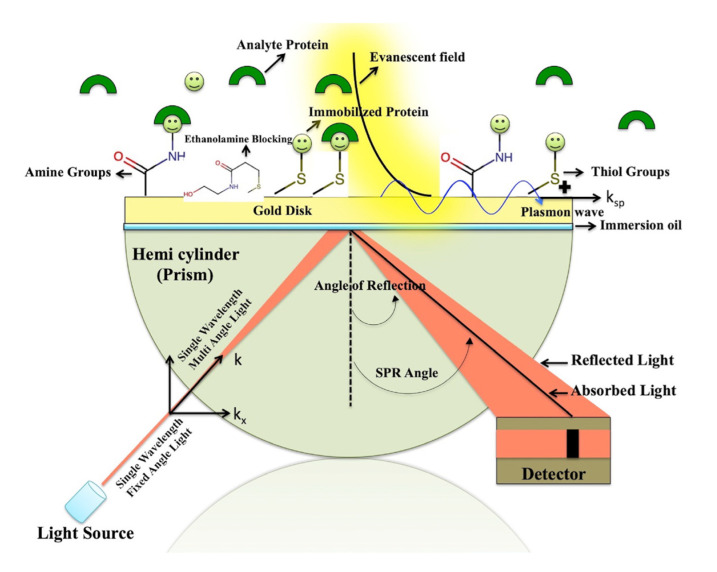
The functionality of SPR biosensors. When the analytes interact with the ligands fixed on the ‘surface’, the dielectric index on the gold ‘surface’ changes, and the reflection maximum is seen at a different angle. This angular shift is the result of the interactions between the analyte and the ligand. (Reprinted with permission [69]. Copyright © 2020, Authors. Exclusive licensee: Bio-protoco1 LLC.)

The light beam propagates in a medium with a larger refractive index 𝑛1 and reaches the common section of a material that has a lower index with a refractive form than the first medium, i.e., 𝑛_2_ (𝑛_1_ > 𝑛_2_). At an incidence angle greater than the limit angle (*θ*), the light is completely reflected and returns to the environment with a higher refractive index [52,53,54,55]. Additionally, no energy is lost during the reflection of the beam, and the light beam causes the penetration of an electric field intensity into the material with a low refractive index, which is introduced as an evanescent wave. The ‘P-polarized’ component of the evanescent subject can penetrate the metal layer and excite electromagnetic surface plasmon waves that are propagated inside the conductive surface associated with a material with a low refractive index if the total internal reflection interface is covered with a layer of appropriate conductive materials, such as metal with an acceptable thickness. This ‘surface plasmon wave’ has P polarization for a non-magnetic metal, namely, ‘gold’, and because of its electromagnetic properties and diffusion surface, it generates an amplified evanescent wave in comparison to incident electromagnetic waves. If the size and directions of the ‘photon wave’ vector 𝑘𝑥 and the plasmon wave vector 𝑘𝑠𝑝 are equal for waves of the same frequency, an amplified evanescent field is produced. When this condition occurs at the landing angle *θ*, the ‘intensity of reflection’ at the angle *θ* can be zero due to the conversion of the energy into a ‘surface’ electric field. With increasing penetration into the thinner material n2, the loss in this evanescent field wave’s amplitude is approximately half the wavelength of its resonance away from the surface. To put it another way, the field loss for visible light is of the order of several hundred nanometers. As a result, just the quenching zone is used to investigate analyte molecules. The SPR biosensor is a group of optical biosensors that have advantages such as real-time detection, a short response time, the simultaneous detection of several types of analytes, and non-labeled sensors [56,57,58,59]. Exciting surface plasmon waves and their characteristics depend on the electromagnetic properties of the dielectric metal interface. Resonance coupling causes a valley in the reflection spectrum at the SPR resonance angle. SPR biosensing can be obtained by the absorption of ‘target analytes’ on the metal surface and dependent changes in the wavelength and intensity in reflected light. These optical changes can rely on alterations in the refractive index due to the phenomenon of surface absorption. Figure 2 shows the basis of SPR biosensors. The high sensitivity to alterations in the features of the dielectric is caused by the transfer of incident light energy to the ‘surface plasmon wave’ and the resulting high density of the electromagnetic field in the dielectric near the metal layer. The gold metal layer’s penetration depth of 200–300 nanometers offers the chance to detect minute variations in the thickness or ‘refractive index’ of layers on the surface of the metal [60,61,62]. The resolution limit of SPR biosensors provides the possibility of detecting surfaces with an approximate coverage of 1 picogram/mm [63,64]. Currently, SPR-based biosensors are the most commercialized type of optical biosensors; they usually have large dimensions and high prices and are suitable for laboratory use [65,66,67]. The technology of surface plasmon resonance sensing as a detector or diagnostic has developed rapidly and has now become an effective tool for direct monitoring and, especially, the analysis of biomolecular interactions. It is also widely used for interactions of biological molecules such as protein–protein supplements, drugs–protein, nucleic acid–protein, nuclear acceptor–DNA, and DNA–DNA. Its fields of application include immunodiagnosis, signal transduction, drug screening, antibody conjugation, and protein conformational changes (Table 1) [56,68].

## 3. SMQDs Structure

**Figure 3:**
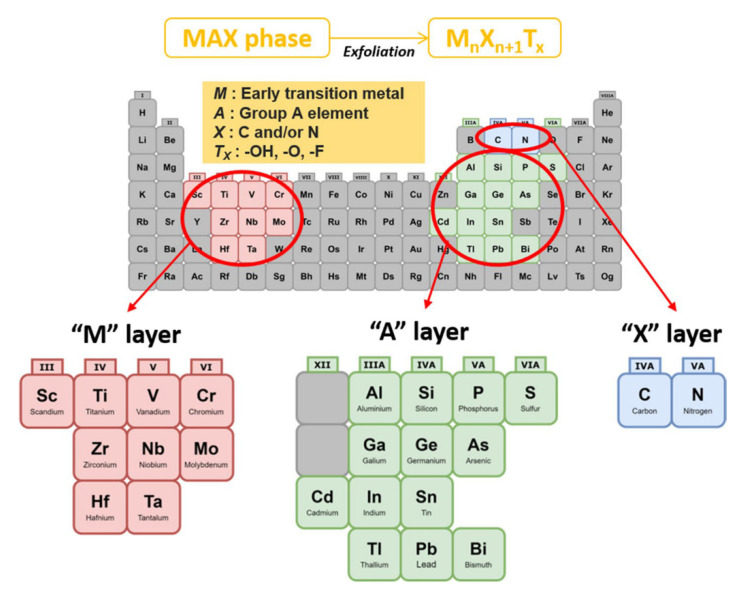
Periodic table of the elements that make up the ‘MAX’ phases as well as SMQDs: M: metals with early transition, I: ‘Group A’ element, X: C as well as Tx, and N: ‘surface function group’. (Reprinted with permission [81]. Copyright © 2021, The Author(s).)

The aging of the ‘MAX -phase’ ‘A’ layers leads to a layered structure of SMQDs, a new class of 2D materials. These MAX phases consist of a large family of nitrides as well as carbonitrides with the chemical formula nAX M_n+1_, where ‘M’ stands for the primary transition metal (such as Sc, Zr, HF, V, Nb, Mo, Ta, Cr) in layer n + 1, ‘A’ stands for an element from the periodic table (usually group 13 or even 14), and ‘X’ stands for carbon as well as ‘nitrogen’ in layers X. [77,78,79,80]. In Figure 3, all constituent elements of the phase MAX are marked with different colors.

**Figure 4:**
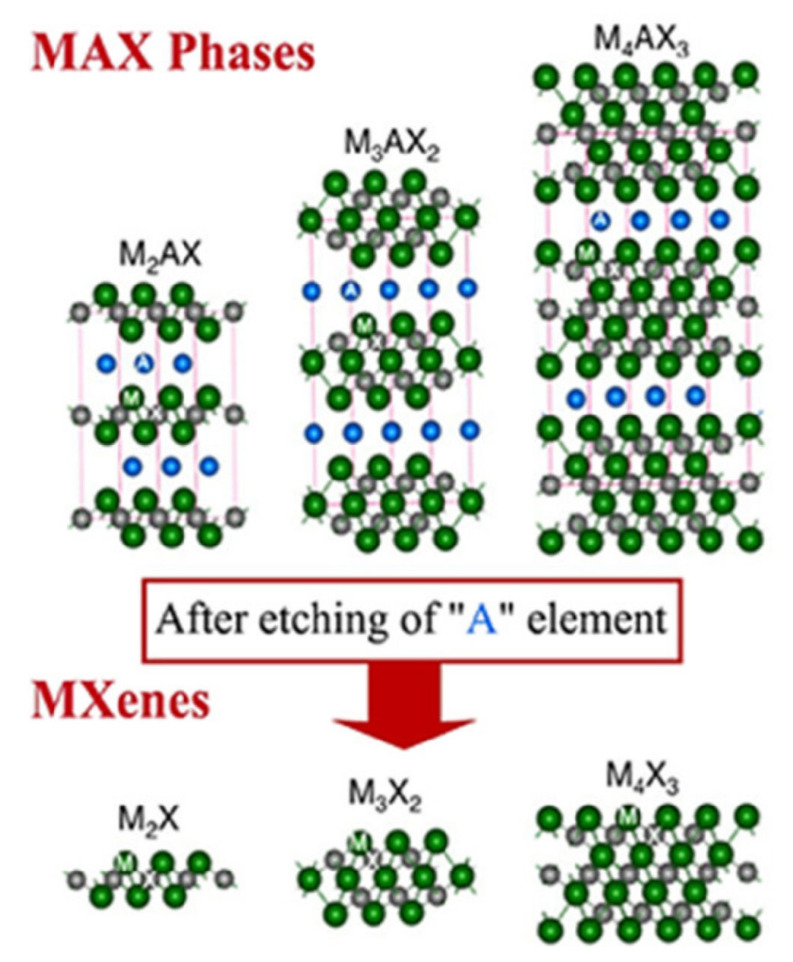
Etching of the MAX phase and creation of SMQDs with surface groups. (Reprinted with permission [99] Copyright © 2021 by the authors. Licensee MDPI, Basel, Switzerland.)

Here, ‘n’ can take the numbers 1, 2, and 3. By changing n from ‘1’ to ‘3’, SMQDs contain layers between three and seven layers of atoms for ‘M2×’, ‘M3 × 2’, and ‘M4 × 3’, respectively [82,83]. As shown in Figure 4, during the etching process, the group A element from the MAX phase is replaced by surface groups such as oxygen (−O), hydroxyl (−OH), and fluorine without destroying the MX layers by suitable chemicals [84,85]. In several studies, Ti_3_C_2_T_x_ with a surface termination group of -Cl has also been observed, and the general formula is M_n+1_X_n_T_x_, where T is the symbol for the surface groups [86,87,88]. MAX has ‘layered’ structures in which the bonds between the layers are weaker than the bonds in the layer [89,90]. In other words, the bonds between M and X are a mixture of ionic and covalent bonds, which can be much stronger than the bonds between M and A [91,92]. As a result, the bond between M and A is decomposed at high temperatures, and the 2D structure M_n + 1_X_n_ is formed. Upon deformation, they become laminated and exhibit a combination of unusual and sometimes unique properties that are intermediate between those of ceramics and metals. For example, like metals, they are capable of conducting electricity and heat, and they can be hard, brittle, and heat-resistant [93,94]. In addition, they are resistant to chemical agents and thermal shocks. However, these ceramics are fabricated as 3D materials, and one of the first experiences with their 2D fabrication is due to ‘2D Ti_3_C_2_’ nanoplatelets. Researchers attempted to remove aluminum from titanium aluminum carbide (Ti_3_AlC_2_) powder by placing it in hydrofluoric acid. Through a chemical process called exfoliation, 2D Ti3C2 nanoplatelets were thus obtained [95,96]. The interesting thing about SMQDs is the naming of this substance. This material is produced from a bulk crystal called Max with the suffix ‘ene’ added to the end, similar to graphene [97,98].

### 3.1. Functional Group on the Surface of SMQDs

Surface end groups that are −OH or −O replace the A layers by the chemical etching of the MAX phase to produce SMQDs. In these materials, two to four M layers are interspersed with layers of C (carbon) or N (nitrogen) in the clever MXene QD structure. Unlike graphene, the surfaces of these materials have functional groups, −O or −OH, that make them hydrophilic. These surface groups are strongly dependent on the etching technique [100,101]. For example, Ti_3_C_2_T_x_ etched with HF has four times more ‘F-functional groups’ than the material etched with a LiF mixture [102,103]. These functional groups also have a great impact on the detection of the electronic functions of SMQDs. For example, it has been shown that both −F and −OH functional groups on the surface of Ti_3_C_2_ lead to a semiconductor behavior with a band gap of 0.05 to 0.1 ev, while Ti_3_C_2_ without surface termination exhibits a metallic behavior [104,105]. Moreover, surface functional groups can influence the energy storage application of SMQDs. For example, density functional theory studies confirm that Ti_3_C_2_ without functional groups stores more lithium ions than its counterpart with a fluorine functional group (Ti_3_C_2_F) because the surface functional groups block lithium adsorption [106,107].

### 3.2. SMQDs Synthesis

The synthesis of SMQDs by precursors is called the top-down method. Depending on the type of precursor, i.e., either MAX or not MAX, this method is divided into two subgroups [100,102]. The most common SMQD precursor is a part of 3D layered carbides as well as nitrides, called the SMQD phase [108,109]. In the precursor materials of the MAX phase, such as M_n+1_AlX_n_ or M_n+1_SiX_n_, various etching methods are used to break the bonds within the layers and replace the individual elements Al and Si with surface groups [110,111]. Layered materials, where the ‘layer-to-layer’ bonding is not significantly stronger than the bonding between layers, are divided into one or more atomic layers to produce 2D materials. Ghidiu and co-workers [107] argued in 2014 that MAX can be etched with a solution of lithium fluoride and hydrochloric acid or with various amounts of hydrofluoric acid. Table 2 lists various approaches for the synthesis of SMQDs. In general, experimental factors such as the etching time, the particle size of the MAX phase, and the acid concentration used affect the better performance in the preparation of higher-quality SMQDs [33]. Recently, some non-Mex phase precursors have been used to fabricate MXs. For example, Zr_3_Al_3_C_5_ has been used as a precursor, although its constituents are similar to those of Al-bonded MAX phase precursors; however, in this precursor, an ‘Al-C’ layer is etched instead of a pure ‘Al’ layer to produce MXene Zr_3_C_2_ [112,113]. Considering that both the constituents of the composition and the surface end groups can be changed, the properties and characteristics of SMQDs can also be easily modified [114,115].

### 3.3. Characteristics and Features of SMQDs

The SMQD material has very interesting properties; for example, although it falls into the category of ceramics, unlike many others, it has good electrical conductivity, which makes it suitable for biomedical applications. The electronic properties of SMQDs are of particular importance because they can be tailored by changing the ‘elemental’ composition of the SMQDs or the surface functional groups. Other factors such as the band gap can also affect the electronic properties of SMQDs. Unlike graphene, SMQD is hydrophilic, which can be very advantageous in many applications. It is also flexible, pliable, and soft. Because of these properties, it can be formed into complex shapes (its use in the form of a tube or a sheet for materials with a conductivity as high as that of metals is very undesirable) [124,125].

### 3.4. Preparing Hybrid NPs Using SMQDs

A SMQDs composite with tiny magnetic Fe_3_O_4_ NPs with a size of about ~4.9 nm (Ti_3_C_2_T_x_/Fe_3_O_4_/TiO_2_) was prepared in an ‘aqueous’ solution of vitamin C and Fe^3+^ salt for 5 h at 150 °C in a stainless steel autoclave with Teflon coating by the hydrothermal method. It is also possible to selectively enrich different biomolecules/antigens based on affinity interactions through these hybrid magnetic NPs. Another interesting alternative for nanocomposites is the combination of SMQDs sheets and metal NPs, which are modified by crosslinkers to detect target molecules due to their strong affinity for SMQDs or other biomolecules [126,127,128]. SMQDs/metal nanoparticle-based nanocomposites can be prepared using an external reducing agent such as NaBH_4_ or the reduction of noble metal salts. To form particles showing surface-enhanced Raman spectroscopy (SERS), the spontaneous reduction of metal salts such as silver, gold, and palladium is applied to Ti_3_C_2_T_x_ SMQDs sheets [129] to form NPs. In addition, it is possible to increase the detection sensitivity of oncomarkers such as microRNA using an AuNP/SMQDs composite [130]. The composite has also been used to detect important small bioactive compounds [131] and electrochemical catalysis [132]. The formation of a composite with SMQDs is also possible using graphite oxide as another 2D material, and such a composite for sensor-based applications leads to the maintenance of the biological activity of hemoglobin even after inkjet printing, as well as the stable and efficient electrochemical detection of H_2_O_2_ [133].

## 4. SPR Biosensors Based on SMQDs

**Figure 5:**
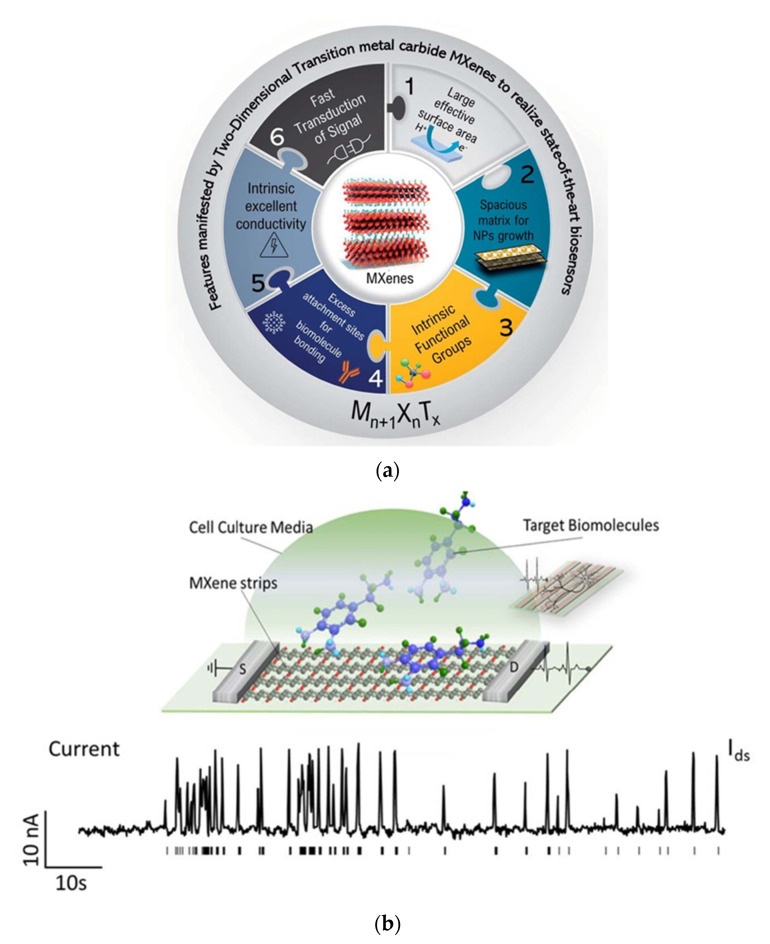
(**a**) Schematic representation of the main features of MXenes with regard to their application in biosensors. (Reprinted with permission [149]. This journal is © The Royal Society of Chemistry 2022.) (**b**) Mechanisms of the SPR biosensors based on SMQDs groups. (Reprinted with permission [150]. Copyright © 2020, Author(s). Published by IOP Publishing Ltd.)

Along with other 2D materials, SMQDs are a potential biosensor application material. In biosensing, the unique benefits of SMQDs include their biocompatibility and minimal cytotoxicity. In addition, MXenes provide a wide adsorption range for optical detection and enhanced DNA interaction [134,135]. MXenes are also related with metallic conductivity, intrinsic surface functionalization, and hydrophilic characteristics, all of which may increase the efficacy of SPR biosensors based on MXenes. The features of MXene that make it necessary for biosensors are summarized in Figure 5a. Ti_3_C_2_, among other compounds, has been extensively documented. Few studies have been conducted on additional MXenes and their composites with metallic nanoparticles, particularly in immunosensing. Ti_3_C_2_, a member of the MXenes and titanium families, is used in a variety of applications, including SPR biosensors. Numerous publications on the diverse uses of Ti_3_C_2_ MXenes in electrochemical and optical smart biosensors have been published [136,137,138,139,140]. Although MXenes have been widely investigated, the biosensor applications of Ti_2_C-MXenes, particularly their composites with nanoparticles, have received less attention (e.g., Au, Ag, etc.). Wang et al. described the production and optical characteristics of Ti_2_C@Au core-shell nanosheets for photonic applications [141]. Zhu et al. built a bifunctional smart nanosensor platform based on Au-Ag nanoshuttles (NSs), utilizing Ti_2_C for the electrochemical and SERS measurement of ultratrace carbendazim (CBZ) residues in tea and rice for environmental monitoring [142]. The mechanisms behind SPR biosensors based on SMQDs usually utilize the exclusive ‘electrocatalytic’ properties of the MXene sheet with respect to the relationship of the ‘target signal’ (Figure 5b) [143]. The electronic properties and current signal change when biological targets are attached to SMQDs films. The 2D layered nanostructure provides a large surface area to accommodate biological materials. The electrocatalytic properties change and lead to a linear response when biological components can be immobilized by functional groups on SMQDs nanocomposites. SPR biosensors built on smart MXene QDs have impeccable repeatability, stability, and reproducibility. The use of functional groups enriched on the surface of SMQDs material could be a potential solution, since non-covalent interactions and physical adsorption are not sustainable for some biomedical applications. This would allow for surface bonding in new and controllable ways to alter surface properties [144,145,146,147,148].

## 5. Advanced 2D SMQDs-Based SPR Nanobiosensors as Ultra-Sensitive Detection Gadgets

**Figure 6:**
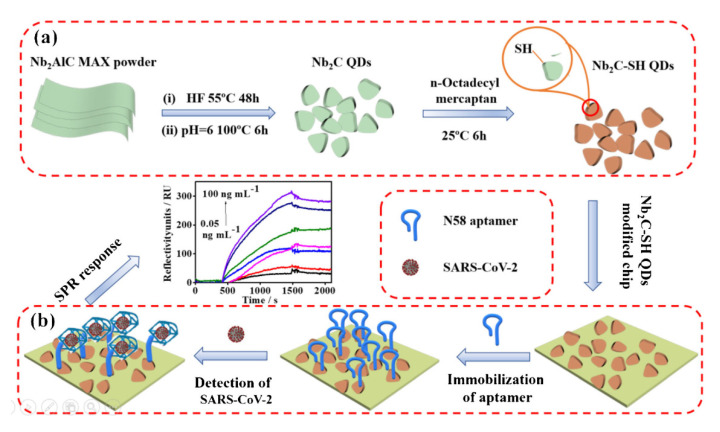
(**a**) Synthesis of Nb_2_C-SH QDs. (**b**) Fabrication of an Nb_2_C-SH QD-based SPR aptasensor for SARS-CoV-2 N-gene detection. (Reprinted by permission [166]. Copyright © 2022, Springer Nature Switzerland AG. Part of Springer Nature.)

The use of biosensing platforms that use nanomaterials or nanostructures with exceptional optical, magnetic, electrical, mechanical, and electrocatalytic capabilities promotes the link between advancing detection and routine testing. Incorporating new multifunctional nanoscale structures, morphologies, and controlled structures and a large surface-to-volume ratio enables immobilization in bioreceptors while maintaining biostability, biocompatibility, and biodistribution [151]. Therefore, the SPR sensing strategy using nanomaterials can not only be used as an effective tool for the detection of difficult-to-detect molecules in the concentration range between pmol and amol, but it also facilitates the improvement of sensing properties [152]. It is expected that the design of SPR biosensors is promising for the ultrasensitive and selective detection of cancer. 2D layered materials such as SMQDs have anisotropic electron transport behavior and a large surface area, which makes them potential transducer materials for biosensing applications [153,154,155]. The results of Wu et al. show an increase in the sensitivity of an SPR biosensor by about 25% with ten graphene layers [156]. Gupta et al. also investigated an SPR biosensor with graphene and silicon to increase the sensitivity [157], and their results showed a maximum sensitivity of ~134.6°/RIU. Ouyang et al. investigated an SPR biosensor with MoS_2_ and silicon to increase the sensitivity [158], and the highest sensitivity was ~125.44°/RIU. Wu et al. investigated a novel SPR biosensor with Ti_3_C_2_Tx-MXene multilayers to increase the sensitivity. According to their results, the sensitivity can reach 224.5°/RIU [159]. SMQDs nanomaterials exhibit a unique combination of excellent mechanical properties, an ease of functionalization, an excellent electrical conductivity, an extremely thin 2D sheet-like morphology, etc. compared to other 2D materials such as graphite carbon nitride, MoS_2_, and graphene [160,161]. Among the properties that significantly affect the strength, sensitivity, and selectivity of a biosensor are the inherent properties of the bioreceptor, including its tendency to be structurally stable during the operation of the biosensor, the analyte, and the method used to stabilize the bioreceptor on the surface of the transducer. The bioreceptor component is often attached to a surface, placing it in close proximity to the transducer. Additional requirements that must be met for improved biosensor performance include the interfacial density of the bioreceptor and the distance between the bioreceptor and the transducer (surface). Aptamers, antibodies, enzymes, and protein molecules can be used to influence the design of biosensors based on 2D SMQDs nanomaterials to improve biocompatibility and increase the transporter surface area of the biosensor in conjunction with the increased activity of the catalyst [162,163,164]. In addition, the implementation of SMQDs as next-generation diagnostic devices requires a significant improvement in the stability of SMQDs against oxidation. Biosensors are small, portable analytical instruments that convert a biochemical process into a quantitative, analytical signal. Because of their high ‘specificity’, small size, and ease of use, biosensors are the preferred instruments for biological components and chemical detection. Biosensors consist of two parts: a bio-detection component that uses a biological element (enzymes, antibodies, nucleic acids, etc.) that interacts with an analyte in a specific biochemical manner, and transducers in which the interaction is converted into quantifiable signals. The integration of the bio-receptor into a suitable matrix for the interaction between analytes and such receptors are the two main obstacles to the improvement of biosensors [165]. Chen et al. designed a new SPR biosensor using thiol-functionalized niobium carbide MXene QDs (referred to as Nb_2_C- SH QDs) as a bio-platform for the N58 aptamer targeting the N gene. As shown in Figure 6, this biosensor was investigated for the sensitive detection of the N gene in various complex environments (e.g., human serum). By the solvothermal method, Nb_2_C-QDs were obtained from Nb_2_C-MXene nanosheets and then modified with thiol groups (Figure 6a). The Nb_2_C- SH QDs were homogeneously distributed on the surface of the chip due to the self-assembly effect between Nb2C- SH QD and the SPR gold chip, and the N58 aptamer was stabilized by hydrogen bonding, π-π* stacking, and electrostatic adsorption. In the presence of SARS-CoV-2, it is also possible to form a G-quadruplex between the N58 aptamer and the N gene of SARS-CoV-2. Thus, upon binding to the N gene, the structure of the aptamer strands is altered, resulting in an increase in the contact area or the distance between the probe molecule and the chip. These changes were then translated into changes in the SPR signal for the detection of the SARS-CoV-2 N gene (Figure 6b) [166].

### 5.1. MXene-Based Electro-Chemical SPR Nanobiosensors

Electro-chemical biosensors can be promising selective tools for detecting cancer diseases in the early stages [167]. SWV (square wave voltammetry), CV (cyclic voltammetry), DPV (differential-pulse-voltammetry), and EIS (electro-chemical impedance spectroscopy) are among the electro-chemical methods [168,169,170]. ‘Lab-on-chip’ biosensors have been miniaturized instruments used in the biomarker research of tumors, leading to potential clinical properties. The small volume of analytes, the direct miniaturization, and the optically absorbing and fluorescent compounds are among the attractive features of biosensors that use surface nano-architectures with this type of detection. Kumar et al. investigated the covalent binding of bioreceptors to f-Ti_3_C_2_ SMQDs for the electro-chemical detection of carcinoembryonic antigen (CEA) as a cancer detector. Single-layer SMQDs (Ti_3_C_2_) nano-sheets were used with 3-aminopropyl tri-ethoxy-silane. The enhancement of antibodies anchoring and faster access to analytes are possible by ultra-thin 2D nano-sheets of single/multilayer Ti_3_C_2_ SMQDs. According to the findings, the synthesized biofunctional Ti_3_C_2_ SMQDs have a linear detection range of 0.0001–2000 n.gm L^−1^, with a sensitivity to approximately 37.9 Ang^−1^ mL cm^−2^ per 10 years [160]. A conductive support for the immobilization of aptamer probes is also employed in 2D SMQDs because of their outstanding electrical conductivity and sizable particular surface areas by a variety of possible binding sites. Lorenkova et al. investigated the electrochemical performance of Ti3C2Tx-MXenes as sensors [162]. The results obtained showed that the detection limit of 0.7 nm is comparable to the best result obtained so far, which is 0.3 nm [171]. However, there are few reports on SPR sensors integrated with MXene. A recent theoretical study of an MXene-based SPR sensor showed that the coating layers on the gold film can increase the sensitivity of the gold-based SPR sensor. An RI sensitivity of 160 was achieved with four layers of coated gold film at an excitation wavelength of 633 nm, while it was 137 for the devoid setup [159,172].

### 5.2. SMQDs-Based Optical SPR Nanobiosensors

An important technique for the in situ detection of the affinity of various biomolecules that do not require enzymatic labeling is SPR. SPR optical sensing technology is also useful for biomolecule detection. To make the SPR optical biosensor specific for the analytes of interest, they need to be functionalized by bio-recognition molecules (such as proteins, RNA, DNA, cells, etc.). The adhesion of biomolecules to the optical surface is generally achieved by chemical bonds such as (3-aminopropyl) triethox-ysilane and N-succinimidyl-4-maleimidobutyrate [173,174,175]. In recent years, 2D transition metal dichalcogenides (TMDs), especially MoS_2_, have attracted the attention of researchers in various scientific fields due to their high optical absorption efficiency, high electron conductivity, and tunable band gap [176,177]. The distinctive features of MoS_2_ that make it a potential material for the development of biosensor interfaces include the presence of free sulfur atoms, its hydrophobic nature, and its large surface area [178,179]. In addition, MoS_2_ layers are also used to inhibit the oxidation of metal layers such as aluminum in SPR biosensors [180]. Additionally, improved operating parameters using nanomaterials have the potential to develop SPR biosensors [181]. The SPR detection platform offers useful advantages such as the ease of miniaturization, ‘label-free’ and ‘real-time’ detections, and rapid detection for bioassays. Ti_3_C_2_T_x_ SMQDs multilayers improve the applicability of SPR biosensors due to their absorption [159]. The ‘gold layer’ SMQDs/WS_2_ ‘phosphorus’-based platform, using a monolayer of each nanomaterial, was shown to be a new SPR ‘sensing material’ with an increased sensitivity of 15.6% compared to bare ‘metal films’ [182]. SMQDs-based composites such as g-C_3_N_4_/SMQDs AgNPs containing g-C_3_N_4_ as a photocatalyst, SMQDs, and AgNPs as electron mediators enhance the photocatalytic activity. In the interface modified with the nanocomposite, the decrease in the band gap energy and the increase in the optical absorption can be observed thanks to the SPR effect of the ‘deposited silver NP’ [183]. The label-free detection of the bovine serum albumin (BSA) protein using an alternative method of fiber optic SPR probe activation with antibodies was evaluated by Kaushik et al. In this new method, gold-coated fibers were first modified with molybdenum disulfide (MoS_2_) nanosheets. The developed technique enables the direct and chemical-free binding of representative antibodies through hydrophobic interactions and also allows for the amplification of SPR signals by the synergistic effects of MoS_2_ and the gold metal thin film. The results showed that the sensitivity of the modified MoS_2_ sensing probe was improved with a detection limit of 0.29 μg/mL compared to the optical fiber SPR biosensor without MoS_2_ coating [184].

**Figure 7:**
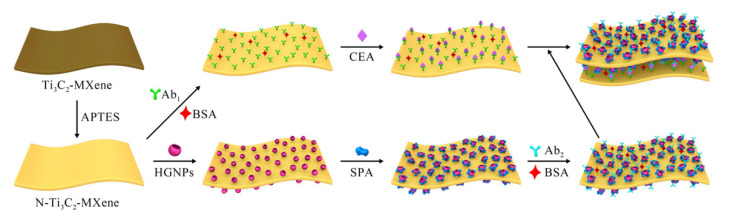
Plan of the prepared ‘SPR biosensor’ detection method. (Reprinted with permission [185]. Copyright © 2020, The American Chemical Society.)

According to Wu et al., employing composites constructed of SMQDs, such as g-C3N4/SMQDs AgNPs, which include g-C3N4 as a photocatalyst, SMQDs, and AgNPs as an electron mediator, increases the photocatalytic activity. The band gap energy is decreased and the optical absorbance is raised at the nanocomposite modified interface as a result of the deposited silver NPs SPR influence. As a signal amplifier, amino-functionalized N-Ti_3_C_2_-MXene-hollow gold NPs (HGNPs)—staphylococcal protein A—were employed for the detection of CEA with a L.O.D of 0.15 fM (linear range of 0.001 to1000 p.M) in SPR (Figure 7) [185].

## 6. Biomedical Applications of SPR Biosensors Based on SMQDs

**Figure 8:**
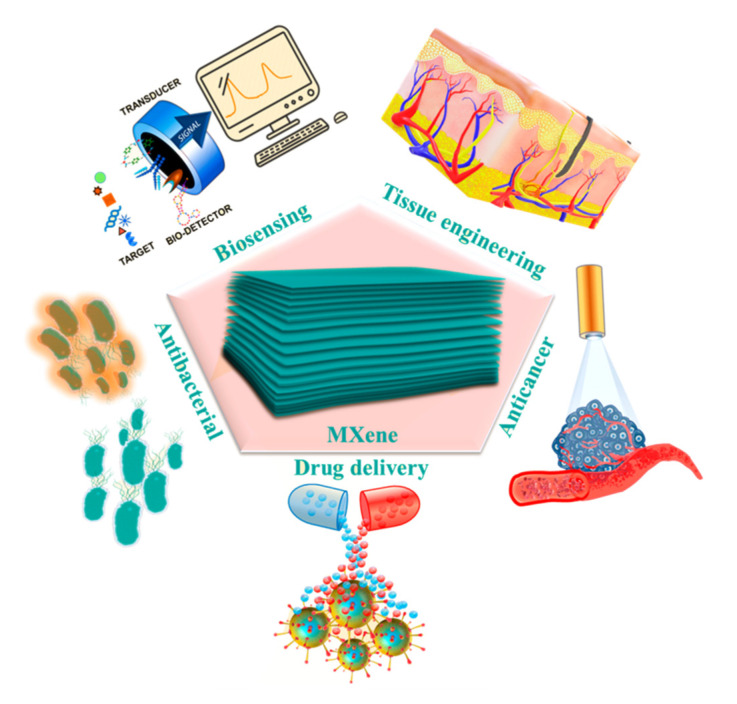
Biomedical applications of SMQDs. (Reprinted with permission [192] Copyright © 2022 by the authors. Licensee MDPI, Basel, Switzerland.)

SPR biosensors provide a label-free, sensitive, specific, and rapid detection method that is preferred for chemical analysis and medical diagnostics [186]. Over the last three decades, since their beginnings in 1982 as gas sensors [187], SPR biosensors based on 2D nanomaterials such as SMQDs have emerged as suitable sensing platforms for a wide range of applications, e.g., in medicine. Various SPR-based configurations have also been investigated for medical and environmental applications, including SPR biosensors based on SMQDs and fiber-optic SPR sensors [188,189,190]. Thus, VDW (Weak van der Waals) forces combined with strong ‘hydrogen bonding interactions’ between ‘surface functional groups’ cause SMQDs to assemble into stacked 2D layers [191]. Chemical reactivity and functionalization ability are among the properties of surface functional groups. In biomedical studies, the level of SMQDs is adapted to various materials suitable for cancer treatment and diagnosis, biosensing, antigen detection, drug delivery, and antimicrobial activity (Figure 8) [81]. The medical applications of SMQDs are shown in Table 3.

### 6.1. Detection of Cancer Biomarkers

SMQDs as new 2D nano-materials have the potential to affect aspects of biosensing such as SPR biosensors in medical applications. Therefore, to detect cancer biomarkers in ‘blood’, SPR biosensors based on SMQDs offer sufficient sensitivity up to ng·m^−1^ or better. In order to simultaneously immobilize biomolecules while resisting non-specific protein binding, much effort should be devoted to finding suitable decoration strategies for SMQDs. These criteria state that, due to their distinct physical and chemical features, SMQDs-based SPR biosensors may be employed to assess complicated substances such as plasma or blood serum (Table 4) [142]. Additionally, the Ti_3_C_2_ MXene-based SPR biosensor in human serum samples exhibits an ultrasensitive cancer biomarker response with a high recovery, good reproducibility, and good selectivity [161]. Additionally, SMQDs with a high density of functional groups have an ultrathin 2D nano-sheet morphology that can optimize biomolecule loading and speed up access to the analyte. In addition to enabling a larger density of bound biomarkers, which improves biosensor performance, the covalent immobilization of bioreceptors including enzymes, DNA, and proteins can also enhance homogeneity and dispersion [199]. Sundaram et al. studied the engineering of MXenes nitrides and 2D transition metal carbides for the therapy and diagnostics of cancer. The findings show that electro-chemical devices based on MXene have the ability to detect cancer biomarkers and have an extraordinarily high sensitivity in identifying the target analyte [200]. To determine the osteosarcoma cancer biomarker by a microgap dielectrode sensor, the MXene surface on multiple connection triangles was investigated. The detection limit and sensitivity were found to be one fM by having good regression co-efficient values (y = 1.0036⨰ +0.525; R^2^ = 0.978), and a current increase was found when raising the target DNA concentration. Based on the results of detecting the levels of operating system complications and the quantification of the survivin gene at a lower level, it can be said that the microgap device with the dielectric surface of multiple connection triangles modified with MXene is useful [201].

### 6.2. Detecting an Exosome as a Supply of Biomarkers of Cancer by Applying 2D SMQDs

**Figure 9:**
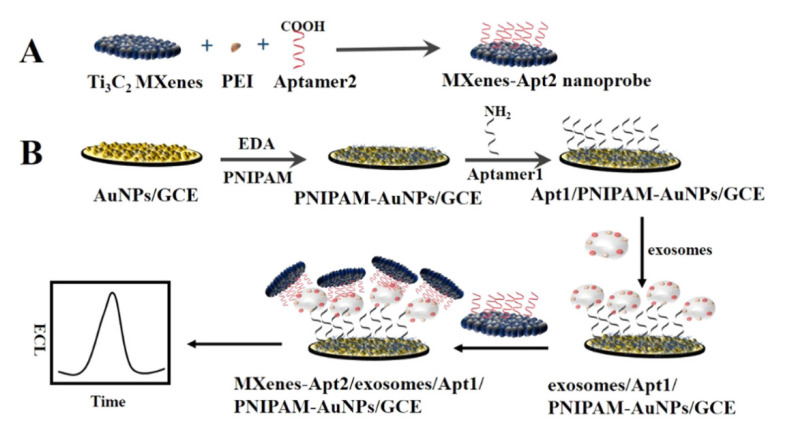
ECL biosensor principle for the signal amplification strategy of exosome activity detection. (Reprinted with permission [207]. © 2018 Elsevier B.V. All rights are reserved.)

Exosome signals transmit in intercellular communications. Additionally, exosomes have the ability to deliver cargo that affects nearby cells and can form pre-metastatic cavities. Exosomes are responsible for the initiation, development, and progression of local malignancies, as well as the formation of metastatic lesions. Exosomes themselves are a popular choice for cancer diagnosis since tumor cells produce more exosomes than normal/healthy cells due to their significantly increased cellular activity [168]. Due to its quick response time, minimal background signal, and high sensitivity, electrochemiluminescence (ECL) has been extensively employed for biomarker research [169]. Because 2D Ti_3_C_2_ MXenes nanosheets have a great conductivity, a large surface area, and catalytic characteristics, Zhang et al. studied the possibility of using them as ECL nanoprobes to create a sensitive ECL biosensor to detect exosomes. The results showed that the limit is about 124 μL^−1^ particles, which is more than 100-fold lower than that of the current enzyme-linked immunosorbent assay (ELISA) method (Figure 9) [207].

### 6.3. Detection of Carcinoembryonic Antigen

Carcino-embryonic antigen (CEA) can be one of the cancer markers considered for cancer diagnosis [161]. Ti_3_C_2_ SMQDs that are monolayer- or multilayer-coated with an acceptor amino group for covalently immobilizing the carcinoembryonic monoclonal antibody for cancer biomarker detection are the first SMQDs-based CEA detectors. SPR technology, which enables chemical molecules as well as refractive index measurements, has been introduced as a technology to increase the sensitivity of the CEA biosensor [185]. However, due to a lack of quick and accurate diagnostic techniques, it is difficult to identify CEA-related tumors at an early stage, which is essential for effective treatment. SPR biosensor technologies can thus be crucial in reaching this objective [171,172]. While ELISA is traditionally used in scientific settings, SPR biosensors based on SMQDs provide a label-free and real-time detection approach. [208]. SPR biosensors according to SMQDs using genetics [209,210] present in tissue have been used for other ailments that take place at high incidence levels [211]. Liu et al. evaluated the detecting growth differentiation factor-11(GDF11) anti-body using an SPR fiber biosensor based on Ti_3_C_2_ MXenes. They found that the detection of GDF11 after activation with the GDF11 antibody is performed by the fiber SPR sensor, and the sensitivity of the fiber SPR sensor increases to 4804.64 nm/RIU. Likewise, the limit of detection in comparison with the single-molecule ELISA procedure could reach 0.577 pg/L, which is 100-fold lower in comparison with that of the single-molecule ELISA procedure [212]. Altintas et al. studied carcinoembryonic antigen cancer biomarker detection. The results showed that a detection limit of 3 ng/mL CEA was achieved with sustainable detections with a correlation co-efficiency of 1 as well as 0.99 for rabbit anti-mouse (RAM) recording assays [213]. Wu et al. used an SPR biosensor based on 2D transition metal carbide MXene for ultrasensitive CEA detection. They also found that Ti_3_C_2_ MXene, as a novel class of 2D transition metal carbides, provides a large compatible hydrophilic surface that is ideal for SPR biosensing. Based on the results, the dynamic range and detection limit for determining CEA is from 2 × 10^−16^ to 2 × 10^−8^ M and 0.07 fM, respectively. Additionally, the results showed that this biosensor approach shows good reproducibility and high specificity for CEA in real serum samples, which provides a promising procedure for evaluating CEA in human serum for the early detection and monitoring of cancer [161].

## 7. Conclusions and Futures Outlooks

In the development of SPR biosensors to achieve high sensitivity and selectivity, numerous nanomaterials have been synthesized and used due to their inherent properties such as extreme conductivity and plasmonic nature. SMQDs attract a lot of attention in developing SPR biosensors due to their exceptional properties. Accordingly, their potential for biosensor development has been widely investigated since the first reports on SMQDs. Moreover, recent research on the improvement of SPR biosensors based on SMQDs has confirmed that, among various nanomaterials, SMQDs may be the best candidates for the development of various types of biosensors, including fluorescent, optical, and electrochemical biosensors. Moreover, there is still much room for progress in the development of SPR biosensor systems and other next-generation biosensors. These views are supported by recent research on the properties of SMQDs and SPR biosensors based on them. SMQDs improve the performance of SPR biosensors and help in the development of SPR biosensors, as explained in this article. The practical application of these SPR biosensors based on SMQDs faces several challenges, such as the reproducibility of these SPR biosensors and their potential for mass production. However, the commercialization of various SMQDs and the development of SPR biosensors based on SMQDs will depend on ongoing research to develop new synthesis techniques or new SMQD architectures. In addition, it is expected that this ongoing research will lead to a more efficient method of combining SMQDs with other nanomaterials to improve the intrinsic properties of new SMQDs that will be developed in the near future.

## Figures and Tables

**Table 1:** Applying surface plasmon resonance biosensors in medical diagnosis.

Field	Detection	Species	Ref.
Medical diagnostics	Virus marker	Ebola, Hepatitis B virus	[70,71]
	Cardiac marker	Myoglobin	[72]
	Drug	Warfarin, Morphine	[73,74]
	Cancer marker	Interleukin 8, Prostate-specific antigen	[75,76]

**Table 2:** A summary of the different SMQDs synthesis methods.

SMQDs	Functionalization(s)	Synthesis Method of SMQDs	Ref.
Ta_4_C_3_	Manganese oxide (MnOx), ‘soy bean’ phospholipid (SP)	HF etching	[116]
Ti_3_C_2_	Poly ‘lactic-co- glycolic acid’ (PLGA), SP, IONPs	HF etching, TPAOH intercalation	[117]
TiO_2_–Ti_3_C_2_	Hemoglobin (Hb), Nafion	Hydrothermal synthesis	[118,119]
Ti_3_C_2_	Cobalt nanowires (CoNWs), Dox	LiF + HCl etching	[120]
Ti_3_C_2_ QDs	–	Hydrothermal synthesis	[121]
Ti_2_N QDs	SP	KF + HCl etching, sonication in NMP	[122]
Nb_2_C QDs	–	HF etching, TPAOH sonication (ultrasoundassisted)	[123]

**Table 3:** The medical applications of SMQDs.

SMQDs	Applications	Ref.
Ti_3_C_2_	Detection of curcumin and hypochlorite (ClO^−^)	[193]
Ti_3_C_2_	Glutathione detection and photoelectrochemical biosensing	[194]
V_2_C Quantum dots	(Bio)imaging, photothermal therapy, and tumor detection	[195]
Ti_3_C_2_	Bioimaging, macrophage labeling, and Cu^2+^ detection	[196]
2D Nb_2_C-MXenes	Photothermal therapy	[197]
Ti_3_C_2_T_x_-SP	Drug delivery	[198]

**Table 4:** SPR nanobiosensor-based SMQDs to detect cancer biomarkers.

MXene-Based Biosensors	Target Biomarker	LOD	Diagnosis Method	Ref.
ssRNA, MoS_2_, AuNPs, Ti_3_C_2_, GCE, and BSA	miRNA-182	0.43 fM	Electrochemical/DPV	[202]
PMo12/PPy@Ti_3_C_2_T_x_/Apt/AE	OPN	0.98 fg m·L^−1^	Electrochemical/EIS	[203]
CD63 aptamer that has been tagged with Cy3 and Ti_3_C_2_ MXenes	Exosomes	1.4 × 10^3^ particles m·L^−1^	Ratiometric fluorescence resonance	[204]
M B, DNA, H T, HP 1, AuNPs, Ti_3_C_2_, BiVO_4_, and GCE	VEGF_165_	3.3 f.M	Photoelectro-chemical	[205]
MXene/IDE HRP-Au-Ab2-PSA-Ab1	PSA	0.031 ng m·L^−1^	Electrochemical/EIS,CV	[35]
N-Ti_3_C_2_T_x_-MXene	CEA	1.7 pg m·L^−1^	SPR	[206]

## Data Availability

All data generated or analyzed during this study are included in this published article.
